# Dr. Carleman’s Portable Russian Vapor Bath Apparatus

**Published:** 1867-10

**Authors:** 


### *Dr. Carleman's Portable Russian Vapor Bath Apparatus.*

Patented, 1867. Manufactured and sold by S. J. Russell & Co. General Office and Salesroom, 196 Lake Street, 2d floor, Chicago. The ingenious inventor will please accept thanks for a specimen of this very convenient and useful apparatus.

